# Asegúrate: An Intervention Program against Cyberbullying Based on Teachers’ Commitment and on Design of Its Instructional Materials

**DOI:** 10.3390/ijerph16030434

**Published:** 2019-02-02

**Authors:** Rosario Del Rey, Rosario Ortega-Ruiz, José Antonio Casas

**Affiliations:** 1 Department of Educational and Developmental Psychology, Universidad de Sevilla, 41019 Seville, Spain; 2 Department of Psychology, Universidad de Córdoba, 41013 Córdoba, Spain; ortegaruiz@uco.es (R.O.-R.); jacasas@uco.es (J.A.C.)

**Keywords:** Asegúrate program, cyberbullying, cyber-victim, cyber-aggressor

## Abstract

This article presents the impact on cyberbullying of the Asegúrate program. This educational program is based on the theory of normative social behavior, self-regulation skills, and the beliefs held by adolescents and consists in a whole package of strategies and resources to help teachers to include in the ordinary curricula. The evaluation of Asegúrate was carried out with a sample of 4779 students (48.9% girls) in 5th and 6th grade in primary education and compulsory secondary education (M = 12.76; SD = 1.67) through a quasi-experimental methodology, with two measures over time. The instrument used was the European Cyberbullying Intervention Project Questionnaire. The results show that the involvement in cyberbullying as cyber-victim, cyber-aggressor, and cyber-bully-victim increase without intervention, whereas it diminishes when intervention is carried out by the teachers who have received specific training and have used the didactic Asegúrate package. Additionally, the impact of the intervention on the different types of behaviors was analyzed, and the results show that Asegúrate is more effective with some forms than with others. Consequently, the Asegúrate program is effective for decreasing the prevalence of cyberbullying, but some modifications need to be made to impact on all the different forms it can take.

## 1. Introduction

In recent years, there has been increased awareness among both teachers and school counsellors that interpersonal relationships between pupils, and the classroom atmosphere in general, are complex processes that are heavily influenced by the social microculture of that particular school and the educational and the pedagogical style of the teaching staff [1]. Until the 1970s, very little attention was paid to certain interpersonal processes, such as the existence of bullying [2]. The increased recognition of this phenomenon has helped to draw more attention to the importance of interpersonal relationships between pupils and how these affect the classroom atmosphere in general [3,4]. The publication and wider circulation of the findings of this research into bullying, and later cyberbullying, has led ordinary school teachers, school counselors, and school staff in general to give more thought to the socio-affective atmosphere in schools, which should be seen as a crucial part of a school’s culture [5]. Over the past thirty years or so, considerable advances have been made in our knowledge of bullying in terms of its prevalence, associated factors, risk indicators, intervention programs, and educational policies [6], and although we cannot yet affirm that all teachers and school counsellors fully comprehend how widespread the problem is, it is clear that they are becoming increasingly aware of its relevance as a factor which harms the healthy balance of welfare and social harmony in schools.

However, since the arrival of internet and, above all, mobile digital technology and social networks accessed via smartphones, social interaction among children and adolescents has become far more complex [7]. Despite the fact that this technological revolution has brought huge benefits in the quality of life of people in many areas of life, such as improved processes of teaching and learning [8], it has also given rise to new problems, such as the evolution of traditional bullying into cyberbullying [9,10]. This poses new challenges to schools, whose job it is to detect and try to prevent this new type of bullying, which while sharing many of the same characteristics as traditional bullying, has other specific features of its own, such as the difficulty for teachers to gain access to these interactions without disturbing the fully justifiable right to intimacy of these children. 

Cyberbullying has been studied from a number of different angles [11], but the most common approach is to deal with it as an extension of traditional bullying “in which the aggression occurs through modern technological devices, and specifically mobile phones or the internet” [12]. This lends it some particular characteristics which are specific to virtual environments and which set it apart it from traditional forms of bullying [13]. Among the features which clearly differentiate traditional bullying from cyberbullying are the possibility that the harassment can take place at any time—24/7, as the well-known phrase goes [14]—and the fact that they are not limited solely to the context of school [15]. Some of the main characteristics of traditional bullying, such as repetition, the power imbalance, and being intentional, are also manifested in slightly different ways in cyberbullying [16]. In cyberbullying, repetition can occur from a single act of aggression if a message or image is sent to many people or forwarded to others [12]. In cyberbullying, the imbalance of power is no longer purely face to face: it can lie in the perpetrator’s greater technological expertise or even to the anonymity afforded to them by virtual environments [17]. Conversely, the idea of intentionality, of knowingly doing harm to others, may not be a factor in some cases of cyberbullying, as many children may be unaware of the negative consequences of sending or forwarding their messages [18].

The prevalence rates of cyberbullying vary considerably from one study to another [19]. According to a recent systematic review including 66 meta-analysis studies and systematic reviews, at least 1 in every 5–7 children is involved in cyberbullying [20]. Similarly, a meta-analysis summarizing 80 studies showed an average rate of involvement of 16% for cyber-aggression and 15% for cyber-victimization, although no figure was given for the dual role of bully/victim [21]. Prevalence rates also appear to vary significantly depending on the way cyberbullying is evaluated and the types of aggression which are included [22,23,24,25]. The few existing longitudinal studies show that the prevalence of cyberbullying is on the rise. It is clear, therefore, that action must be taken against this growing problem, which is producing such serious consequences [26].

This scientific evidence about the existence and consequences of cyberbullying, together with alarming stories of cyber-victims, which appear in the media and its close links with traditional bullying [27,28,29] have led to the measures and programs previously used against bullying being adapted to educate children against cyberbullying, to try and stamp it out or, at least, alleviate its effects [30]. The ViSC Social Competence Program [31], for instance, was originally designed to counter traditional bullying and has been shown to have some effect on cyberbullying, although the authors themselves have admitted that a greater impact would be achieved if specifically designed programs were used. Some programs to prevent and counter cyberbullying have in fact already been designed and evaluated positively. In all, eight programs have been evaluated to date; however, they all seem to be more effective against cyber-victimization than cyberbullying [32] and specific training processes are required which demand an extremely high degree of involvement on the part of the teaching staff, even before the intervention program with the pupils takes place [33].

It has been shown that the teaching staff play a vital role in the effective implementation of programs against traditional bullying [34], and the knowledge and experience they have in dealing with past cases of bullying are particularly valuable [35]. We therefore consider it crucial that teachers gain direct, hands-on experience in introducing anti-cyberbullying programs, which also allows the programs to reach a greater number of people in the educational community and, as a result, obtain more effective results [36]. In the same way, the more directly teachers are involved in analyzing cyberbullying and devising strategies to cope with it, the better grasp they will have of the phenomenon [37], thus responding to the need for teachers to feel better informed and more professionally prepared for this task [38]. In fact, teachers and school principals already consider themselves key players in the prevention and management of all types of cyberbullying [39], despite the fact that their knowledge may be limited to its more widely-known forms [40].

Unlike other research projects in which teaching staff have been trained intensively or extensively to participate in implementing programs against cyberbullying [41], the Asegúrate program has been designed for use by teachers without the need to take part in costly training processes [42]. The training of teachers is of course extremely important in any area of action, but it is also our aim that every school, regardless of its means, has the chance to run a program against cyberbullying. We hope, therefore, that the Asegúrate teaching material will serve as a useful tool for schools, which lack the necessary resources to run specific training courses. The Asegúrate teaching materials consist of a Teacher’s Manual, worksheets for presenting the program in the classroom, and a guide to working together with the children’s families, which uses materials designed to raise awareness.

### The Teacher’s Manual: A Key Component of the Asegúrate Program 

The Asegúrate program rests on three theoretical pillars: the theory of normative social behavior, the principles of constructivist methodologies, and the development of self-regulation skills. The first of these highlights how social behavior is significantly influenced by three normative mechanisms: group identity, expectations, and recognized legal norms. It upholds the notion that our behavior is likely driven by what is perceived as socially acceptable, normal, and legal [43,44]. The constructivism principles are concreted on the fact that each session starts with the exploration of young people’s own ideas and beliefs [45]. As regards the development of self-regulation skills, Asegúrate includes reflective activities aimed at enhancing metacognitive skills to develop strategic learning among students [46]. Further details can be obtained by reading Del Rey et al. [47].

The complete program consists of eight sessions on cyberbullying and other related factors, including the ways people communicate on social networks and their implications; anomalies in online behavior; criteria for establishing safe online friendships; cybergossip; sexting; the abuse of the Internet and social networks, and the norms of cyber-etiquette. Detailed instructions are given as to how to conduct the tasks for each of the eight sessions in the program. Each session contains a specific activity that ensures that the requirements of the Asegúrate methodology are fully complied with. Each teacher is therefore given a full description of the steps to follow with their pupils, and extra resources and explanations are included. Finally, there is a self-access reference section including a glossary of terms, a resource bank (such as descriptions of the most popular YouTubers), links to further reading, etc. Another section provides the answer keys and instructions for evaluating each session and the Asegúrate program as a whole. Apart from the manual, the Asegúrate program also features audio-visual material for each of the sessions for teachers to use with the pupils and their families, as well as awareness-raising publicity resources such as posters, stickers, or bookmarks. The idea is for the teachers to use this complete range of resources to help them tackle the problem of cyberbullying with their pupils.

The Teacher’s Manual fulfils the basic requirements as a useful teaching guide for professional adults to learn autonomously and provides both general guidelines and detailed procedures, including thorough instructions for the tasks and roles, self-help, and specifically targeted orientation [48]. The orientation consists of clear, concise information about the current situation of cyberbullying, a description of the program, and a summary of the methodology used, which is one of the key features of the program. All the Asegúrate presentation sessions are designed with a similar sequence of learning stages, planned in such a way that all participants can work together to understand the key issues. This sequence of activities is made up of five stages, named in the following way, to echo the activities which young people perform on social networks: (a) “Trending topic” explores the current ideas held by the participants; (b) “My profile” encourages the participants to reflect on any of their own activities on the social networks which might be described as “not normal”; (c) “Stop to think” focuses on analyzing the reasons which lead us, or others, to behave in certain ways when using social networks; (d) “Like/Don’t like” identifies the possible consequences of their own (and others’) positive or negative behavior on social networks; and (e) “I share” allows each session to end with a conclusion and an individual and/or collective declaration of commitment.

Taking all of the above into account, the aim of this study was to evaluate the effectiveness of the Asegúrate program in counterbalancing, through education, the involvement of schoolchildren in cyberbullying, whether in the roles of victim, aggressor, or in the dual role of cyberbully/victim, as well as stemming the increase in cyber-victimization and cyber-aggression both in general and specific cases of aggression.

## 2. Materials and Methods 

This study has a longitudinal, quasi-experimental design. There are two study groups—a quasi-experimental group and a quasi-control group—and two data collection points—a pre-test before the program and a post-test at the end of the program.

### 2.1. Participants

A total of 4779 pupils (48.9% girls) took part in this study, from the 5th year of Spanish primary education (average age: 10–11) to the 4th year of Spanish secondary education (average age: 15–16 or higher), from 18 different schools. The ages ranged from 10 to 18 years old (M = 12.76, SD = 1.77). The experimental group consisted of 2316 pupils (50.4% girls) from 18 schools, and the control group contained 2463 pupils (47.5% girls) from 10 schools. The sampling was incidental: access was through invitations issued to schools to participate in the program, and the schools could agree whether to accept or not.

### 2.2. Questionnaire 

The European Cyberbullying Intervention Project Questionnaire (ECIPQ) was used to assess the level of involvement in cyberbullying [49]. This scale was composed of 22 items and contained two different dimensions, assessing the frequency of cyber-victimization and cyber-aggression over the last two months. The responses were Likert-type (0 = No, 1 = Yes, once or twice, 2 = Yes, once or twice a month, 3 = Yes, about once a week, 4 = Yes, more than once a week). Examples of the items for cyber-victimization included the following: *Someone has posted threatening messages against me on Internet*, *social networks, or WhatsApp.* Examples for cyberbullying included the following: *I have insulted someone through social networks or WhatsApp*. The reliability of the scale for the present study was α = 0.80 (α = 0.76 for cyber-victimization and α = 0.73 for cyberbullying).

### 2.3. Process

The schools were contacted by phone and asked if they would like to take part in the study. An appointment was arranged for those schools which agreed, and the schedules and the classes which would participate in the study were decided. With the agreement of the teaching staff, the questionnaires were administered during class time by staff in training, who were specially instructed for this task. Before answering the questionnaires, the participants were fully briefed about the voluntary nature of participating in the study, the anonymity and confidentiality of the data, and the importance of giving truthful answers.

After the data was collected in February 2017, the Asegúrate program was carried out in the quasi-experimental groups, but not in the control groups. After the program was completed, the questionnaires were repeated, at least three months after they were first answered, June 2017 at the latest. Those schools where the program was not carried out were offered the chance to take part in it once the study was completed.

The research was carried out following the ethical standards agreed on by each schools’ Parents’ Association, and was approved by the Andalusia Biomedical Research Ethics Coordination Committee (0568-N-14), which adheres to the guidelines of the International Conference of Good Clinical Practice. All the materials and questionnaires that made up the project were presented and explained to the school management, which gave it their approval in all cases. They, in turn, discussed it with the School Council, as part of the schools’ Projects for Peaceful Coexistence and School Improvement Plans; again, full approval was given for the schools to take part in the study.

### 2.4. Analysis of Results

The first step in analyzing the results of this research was to check the response frequencies for the different types of behavior evaluated by the questionnaire. Next, the prevalence of involvement was calculated following the criteria proposed by the authors of the scales used [49]: pupils were considered “victims” if they answered “*once or twice a month*” or more to any of the questions about victimization behavior and simultaneously answered “*no*” or “*once or twice*” to questions regarding all behavior related to aggression. To calculate the level of aggression, pupils were considered “bullies” if they stated that they had harassed someone “*once or twice a month*” or “more frequently” for any type of behavior classed as cyberbullying and simultaneously answered “*no*” or “*once or twice*” for types of behavior linked to cyber-victimization. In the same way, pupils were labeled “bully/victims” if they answered “*once or twice a month*” or more for behavior linked to victimization and aggression.

The percentage variation was then calculated for each of the groups (control group and quasi-experimental group). This variation shows the difference in prevalence between the values shown in the pre-test and those in the post-test. The following formula was used to calculate this variation: [(PrevalenceT2 − PrevalenceT1)/PrevalenceT1] × 100.

Finally, to evaluate the effectiveness of the program for the different types of behavior in the study, the linear mixed model or repeated measures MANOVA was calculated.

## 3. Results

Firstly, the descriptive results were calculated by examining the frequency of responses in both groups (experimental and control) for the different types of behavior measured by the questionnaire, in order to evaluate involvement in cyberbullying in the pre-test (see Table 1). The most important finding was that the most prevalent type of behavior in both groups, both for victimization and for aggression, was that which was most similar to traditional bullying (insults, threats, social exclusion, and spreading rumors). On the other hand, the types of behavior found more commonly in virtual environments, such as identity theft or re-editing images or videos, were much less frequent (see Table 1).

Next, we calculated the percentages of involvement in the different roles of cyberbullying (victims, aggressors and bully/victims), for both the experimental and control groups at both the pre-test and post-test stages (see Table 2). Here, the roles of victimization, aggression, and bully/victim were notably lower in the experimental group, compared with a slight decrease in number of victims and aggressors and a marked increase in the number of bully/victims in the control group.

Finally, the repeated measures MANOVA was calculated for each of the 11 items of victimization and aggressive behavior measured in the cyberbullying questionnaire at the two stages when these were carried out (see Table 3). These results show significant overall differences in the level of victimization, with a clear decrease in the experimental group and no changes in the control group (F_(1, 4507)_ = 12.63, *p* < 0.01, d = 0.29). The same pattern could be observed for the level of aggression, where the control group score remained the same, while there was a significant fall in the score in the experimental group after the program was carried out (F_(1, 4526)_= 6.66, *p* < 0.01, d = 0.25).

As regards the specific behavior of victimization, after the program was carried out, direct insults (F_(1, 4507)_ = 4.05, *p* < 0.05, d = 0.20), posting insults about the victim (F_(1, 4507)_ = 5.16, *p* < 0.05, d = 0.21), threats (F_(1, 4507)_ = 5.25, *p* < 0.05, d = 0.21), exclusion (F_(1, 4507)_ = 15.75, *p* < 0.01, d = 0.30), and spreading rumors (F_(1, 4507)_ = 9.42, *p* < 0.01, d = 0.25) decreased clearly in the experimental group compared to the control group, which showed either no changes or a slight fall.

As regards aggressive behavior, the scores for insults (F_(1, 4526)_ = 14.66, *p* < 0.01, d = 0.30), threats (F_(1, 4526)_ = 5.87, *p* < 0.01, d = 0.22), and exclusion (F_(1, 4526)_ = 7.21, *p* < 0.01, d = 0.23) decreased significantly in the experimental group compared to the control group, which saw hardly any changes or even a slight increase.

## 4. Discussion

The aim of this study was to assess the impact of Asegúrate, an educational program specifically designed to prevent, reduce, or alleviate cyberbullying, in which teachers were given a purpose-written self-training manual (Asegúrate) to use autonomously. This educational material focuses on cyberbullying and on the social behavior teenage boys and girls display online when using virtual social networks. The Asegúrate program assumes that teachers are sufficiently aware that this behavior can disrupt the social climate and well-being of their pupils and that they will carry out self-training and acquire suitable levels of professional competence to intervene when such behavior occurs. We also assumed that, if teachers increased their competence in this way by using the materials available to them including the Asegúrate manual, then the levels of cyberbullying in the classroom would improve, as measured by self-administered questionnaires given to the schoolchildren themselves, as is the norm in studies of bullying and cyberbullying [50]. This system of pre/post evaluation has enabled us to note any improvements in prevalence for the different aspects of cyberbullying/cyber-victimization evaluated with the questionnaires, and to rank the results in order of highest to lowest prevalence of the different types of cyberbullying. 

This has allowed us to observe that verbal cyberbullying, which is close to traditional forms of bullying, such as insults, threats, social exclusion, and spreading rumors, is the most frequent type in both groups, both in victimization and aggression: in other words, the commonest form of cyberbullying is still very similar to traditional bullying. By this, we can deduce that the new forms of cyberbullying introduced by technology, such as identity theft or manipulating multimedia, images, or videos with the intention of offending or hurting the victim, are less widespread among the adolescent population, at least in the cross section of pupils who participated in this study. These results also seem to show that the main aspect that is transferred to the online context is traditional aggressive behavior, to a far greater degree than the actual use of technology. We feel this is a relevant discovery, since it supports the idea that rather than the digital platforms opening the floodgates to new kinds of bullying [51], cyberbullying mainly consists of simply adapting the usual offensive roles of bullying one’s peers to the conditions provided by the current technology [27].

In addition, the secondary objective of this work was to evaluate the effectiveness and suitability of the self-training manuals written by our scientific team and made available to the teachers who took part in the study (the experimental group) to use as they saw fit. The Asegúrate teaching manual, interpreted and used autonomously by teachers in the schools involved in the study, has led to significant changes in the pupils’ involvement in the program. The experimental group obtained extremely positive results in improving the situation regarding victimization, aggression, and the number of bully/victims. Some improvement was also noted in the control schools, but to a lesser extent than in those involved in the experiment. The most notable result was the clear decrease in victimization, aggression, and bully/victims in the experimental group, compared to the very slight fall for the control group in victims and aggressors and the sharp rise in bully/victims [52]. If these results are interpreted correctly, there is a higher risk of becoming bully/victims in those schools where no intervention program (i.e., Asegúrate) is applied. In other words, the rise in aggressiveness and victimization was the greatest risk in cases where the teachers did not specifically take part in self-training and become aware of how they could use their own professional skills to prevent the problem. When comparing the post-tests for the control group, the findings show an increased level of conflict from bully/victims. 

The role of bully/victim, in which pupils are not only victimized by their peers, but are also aggressors at the same time, has been described as one which involves far greater problems of psychological adjustment [53]. Although there are few existing longitudinal studies on the subject, the Asegúrate program has proved effective in dealing with the phenomenon where cyberbullying leads to a higher incidence of this double role of involvement [54]. It has been shown that, when no action is taken—apart from other insignificant, circumstantial changes that cannot be measured objectively—the number of pupils who adopt both roles increases. The increased number of pupils involved in this controversial mixed role (bullying others as well as being a victim of bullying) could be considered as an underlying, often unnoticed, result of not acting against cyberbullying. We could say that, if the problem is ignored, and no action is taken, it results in a greater, indiscriminate risk of increased conflict, aggression, and victimization, which is possibly the most confusing and chaotic scenario produced by bullying and victimization.

When both experiences decrease (cyber-victimization and cyberbullying), the involvement of teachers can become an extremely relevant factor for change, since the phenomenon of cyberbullying is mutualistic, in that bullying is both given and received. Teachers who make it a priority in the course of their professional activity to focus on specific problems of bullying and deal with them effectively, guided by their own convictions, appear to exercise a strong influence against the spread of cyberbullying [55]. The Asegúrate program prioritizes professional autonomy and effective management, and the results obtained show good efficacy in terms of reducing cyber-victimization and cyberbullying. After the program was introduced, direct insults, abuse, or verbal aggression against the victim, threats, social exclusion, and rumor-spreading all diminished significantly. This clearly shows that, in groups where the teachers implemented the program, the incidence of threatening, insulting, and socially excluding behavior fell sharply. In particular, the repeated measures results for each of the 11 items of behavior of cyber-victimization and cyberbullying showed that the Asegúrate program proved particularly effective against the kinds of virtual bullying and victimization, which are most similar to face-to-face bullying: verbal abuse, and social and psychological exclusion. Bullying involving insulting and humiliating decreased in those schools which took part in the study, while it remained the same in the control schools. This decrease was significantly greater in the schools taking part in the study than in the control schools for bullying involving verbal abuse, victimization, and social exclusion. This program therefore seems to have fulfilled its primary objective, which was to offer teachers the necessary resources to take a stand against cyberbullying in their schools, a demand that has been so widely called for by the scientific community [56]. 

After the initial self-training, the teaching staff who put the Asegúrate program into practice made special efforts to combine activities specially designed by experts with their own interpretation and self-planned techniques, which they adapted to the social context of their own pupils. This illustrates the importance of the teaching staff as a key element in the intervention against cyberbullying [39]. Just as happens in traditional bullying, schoolchildren see the degree of teacher involvement as a highly relevant factor in reducing or facilitating their involvement [57]. It has also become clear that the extent to which pupils perceive that their teachers are involved in managing interpersonal relationships in the classroom has a high predictive value for cyberbullying. This reinforces the idea that, when the pupils themselves recognize high quality teaching, it includes the notion that this high quality both helps to prevent cyberbullying as well as improve the general atmosphere of the class [58].

The Asegúrate program has been effective against all the types of behavior, which are associated with involvement in cyberbullying, which represents such a problematic phenomenon for schools. In fact, the indicators which show a reduction of involvement in both roles reveal highly significant changes. Not only does the specific behavior of cyberbullying and cyber-victimization decrease, but also a large number of the boys and girls who were previously noted as “involved in cyberbullying phenomena” in the pre-test were classified in the post-test in the “not-involved” or “bystanders” group. In fact, there was a significant increase in the number of “bystanders” in the post-test in the experimental group compared with the control group, with around a 25% decrease in the number of cyber-victims and a fall of over 30% in the number of cyberbullies.

It should also be noted that the evaluation of the Asegúrate program has certain limitations. The use of self-reports has its obvious shortcomings, but perhaps the most relevant is that the Asegúrate program could be improved further with more specific training to deal with the specific behavior of schoolchildren when using digital devices. Although this type of behavior does not appear to be extremely widespread, the harmful, dangerous behavior it involves did not decrease in adolescents even after the program was carried out. It therefore seems essential to include a study of the dynamics of how adolescents use digital devices, as well as materials and training to help make the program more effective in this respect. Likewise, other variables that are impossible to measure in this type of research could be related to the changes found in other activities in the school, additional teacher training, etc.

## 5. Conclusions

The results we obtained are in line with the different articles in the scientific literature about the prevention of cyberbullying, which show that the teaching staff are one of the key factors in reducing and alleviating the problem [16,20,59]. However, our results go one step further: they show how effective material, designed by researchers who have thorough knowledge of the prevalence and characteristics of cyberbullying, used autonomously by teachers who know their pupils well and spend long periods of time in the classroom with them, is a highly effective and beneficial combination, because it does not disturb the normal daily life of the classroom. When intervention programs such as the one proposed in this research are put into practice, the autonomy and initiative of the teaching staff is viewed by the pupils in a positive light, as a vital means of support. Each teacher may adapt the material to suit the needs of their school and any particular class, but the practice of using common guidelines, such as those provided by the Asegúrate teaching material, has been widely accepted. This does not mean that external training processes are no longer necessary: on the contrary, this external support for their teaching helps the autonomous training provided by each teacher to be both more effective and better appreciated by the schoolchildren.

Although cyberbullying occurs in a virtual environment, where some teachers feel less comfortable or reluctant to explore because of their lack of prior experience [60], our results show that the most common behaviors are those more like traditional bullying ones. These types of behaviors through electronic device have been the most reduced by teachers, perhaps because they have previous experience in carrying out anti-bullying programs. 

For this reason, our results emphasize that it is not essential that teachers immerse themselves or have an expert domain of virtual environments, social networks, or cross-platform applications to improve how their students behave online. The useful work that has been carried out over a long period of time against traditional harassment [61], which promotes values such as empathy and respect for colleagues, can be developed and transferred to the context of virtual environments, with the help of high quality teaching materials, such as those provided by the Asegúrate program.

## Figures and Tables

**Table 1:** Descriptive results calculated by groups experimental and control.

Items	0: No		1: Yes, Once or Twice		2: Yes, Once a Month		3: Yes, Once a Week		4: Yes, More Times a Week	
	% Exp	% Contr	% Exp	% Contr	% Exp	% Contr	% Exp	% Contr	% Exp	% Contr
Someone said nasty things to me or called me names using Internet, social networks, or WhatsApp.	72.6	79.9	22.2	16.3	2.7	2.4	0.7	0.6	1.7	0.9
Someone said nasty things about me to others using Internet, social networks, or WhatsApp.	78.7	84.2	17	12.6	2.3	1.9	0.8	0.6	1.3	0.7
Someone threatened me through texts or online messages.	88.1	92.5	10.1	6.5	0.9	0.7	0.3	0.2	0.6	0.1
Someone hacked into my account and stole personal information (e.g., through email or social networking accounts).	94.2	96	5.3	3.7	0.2	0.1	0.1	0.1	0.2	0.1
Someone hacked into my account and pretended to be me (e.g., through instant messaging or social networking accounts).	93.7	95.7	5.7	3.4	0.2	0.5	0.2	0	0.1	0
Someone created a fake account, pretending to be me (e.g., through instant messaging or social networking accounts).	95.1	96.3	4.4	3.3	0.2	0.3	0.1	0	0.2	0
Someone posted personal information about me online.	93.6	94.2	5.5	5.5	0.4	0.1	0.2	0	0.3	0.2
Someone posted embarrassing videos or pictures of me online.	90.9	93	7.4	5.9	0.7	0.6	0.5	0.3	0.5	0.3
Someone altered pictures or videos of me that I had posted online.	93.1	95.1	6.1	4.3	0.6	0.3	0.1	0.1	0.2	0.1
I was excluded or ignored by others in a social networking site or Internet.	80.6	86.6	16.5	12.1	1.5	0.9	0.4	0.2	0.9	0.2
Someone spread rumors about me on the Internet.	85.4	88.6	12.2	9.7	1.3	1.2	0.5	0.1	0.6	0.3
I said nasty things to someone or called them names using Internet, social networks, or WhatsApp.	79	83.6	17.9	14	1.6	1.7	0.9	0.3	0.7	0.4
I said nasty things about someone to other people using Internet, social networks, or WhatsApp.	82.6	85.5	14.6	12.3	1.4	1.4	0.8	0.5	0.7	0.4
I threatened someone through texts or online messages.	94.4	97.1	4.7	2.3	0.5	0.3	0.1	0.2	0.3	0
I hacked into someone’s account and stole personal information (e.g., through email or social networking accounts).	97.8	98.9	1.7	1.1	0.3	0	0	0	0.2	0
I hacked into someone’s account and pretended to be them (e.g., through instant messaging or social networking accounts).	98.2	98.8	1.4	1.1	0	0	0.1	0	0.3	0.1
I created a fake account, pretending to be someone else (e.g., through instant messaging or social networking accounts).	97	97.5	2.7	2.1	0.1	0.2	0.2	0.1	0.1	0
I posted personal information about someone online.	97.9	98.4	1.7	1.3	0.3	0.1	0.2	0.1	0	0
I posted embarrassing videos or pictures of someone online.	96.5	96.6	2.8	3	0.3	0.2	0.2	0.1	0.2	0.1
I altered pictures or videos of another person that had been posted online.	95.2	96.3	4.4	3.1	0.2	0.2	0.2	0.2	0.1	0.1
I excluded or ignored someone in a social networking site or Internet.	85.8	90.4	12	8.5	1	0.7	0.2	0.1	1	0.2
I spread rumors about someone on the Internet.	93.9	95	5.1	4.3	0.3	0.4	0.3	0.2	0.3	0

**Table 2:** Percentages of involvement in the different roles of cyberbullying.

	% Not Involved	% Victims	% Aggressors	% Bully-Victims
PreExperimental	83.9	7.9	4.5	3.7
PostExperimental	87.5	6.0	3.1	3.4
PreControl	88.1	6.4	3.0	2.5
PostControl	88.5	6.0	2.5	3.0
%Experimental Change	4.29	−24.05	−31.11	−8.10
%Control Change	0.45	−6.25	−16.66	20

**Table 3:** Multivariate analysis of variance (MANOVA) results.

Items	Group	M (SD)		Lambda de Wilks F	*p*
		Pre	Post		
General Victimization	Exper	0.15 (0.25)	0.12 (0.25)	12.63	≥0.01 *
	Control	0.10 (0.19)	0.10 (0.22)		
General Aggression	Exper	0.09 (0.18)	0.06 (0.19)	6.66	0.01 *
	Control	0.06 (0.15)	0.06 (0.16)		
Someone said nasty things to me or called me names using Internet, social networks, or WhatsApp.	Exper	0.37 (0.73)	0.33 (0.67)	4.05	0.04 *
	Control	0.26 (0.61)	0.26 (0.62)		
Someone said nasty things about me to others using Internet, social networks, or WhatsApp.	Exper	0.29 (0.67)	0.25 (0.60)	5.16	0.02 *
	Control	0.21 (0.56)	0.21 (0.56)		
Someone threatened me through texts or online messages.	Exper	0.15 (0.48)	0.13 (0.46)	5.25	0.02 *
	Control	0.09 (0.34)	0.10 (0.41)		
Someone hacked into my account and stole personal information (e.g., through email or social networking accounts).	Exper	0.07 (0.31)	0.07 (0.36)	1.17	0.29
	Control	0.05 (26)	0.06 (0.30)		
Someone hacked into my account and pretended to be me (e.g., through instant messaging or social networking accounts).	Exper	0.07 (31)	0.07 (0.37)	0.57	0.56
	Control	0.05 (0.23)	0.05 (0.29)		
Someone created a fake account, pretending to be me (e.g., through instant messaging or social networking accounts).	Exper	0.06 (0.29)	0.06 (0.31)	0.80	0.36
	Control	0.04 (0.22)	0.05 (0.26)		
Someone posted personal information about me online.	Exper	0.08 (0.36)	0.07 (0.32)	0.35	0.55
	Control	0.07 (0.29)	0.06 (0.30)		
Someone posted embarrassing videos or pictures of me online.	Exper	0.12 (0.45)	0.09 (0.38)	0.77	0.38
	Control	0.09 (0.38)	0.07 (0.33)		
Someone altered pictures or videos of me that I had posted online.	Exper	0.08 (0.33)	0.07 (0.32)	0.52	0.47
	Control	0.06 (0.28)	0.05 (0.27)		
I was excluded or ignored by others in a social networking site or Internet.	Exper	0.25 (0.59)	0.16 (0.48)	15.75	≥0.01 *
	Control	0.15 (0.43)	0.13 (0.43)		
Someone spread rumors about me on the Internet.	Exper	0.19 (0.52)	0.12 (0.42)	9.42	≥0.01 *
	Control	0.14 (0.43)	0.12 (0.45)		
I said nasty things to someone or called them names Internet, social networks, or WhatsApp.	Exper	0.27 (0.61)	0.19 (0.53)	14.66	0.00 *
	Control	0.20 (0.51)	0.19 (0.52)		
I said nasty things about someone to other people using Internet, social networks, or WhatsApp.	Exper	0.23 (0.57)	0.17 (0.52)	1.4	0.23
	Control	0.18 (0.49)	0.14 (0.44)		
I threatened someone through texts or online messages.	Exper	0.07 (0.34)	0.05 (0.28)	5.87	0.01 *
	Control	0.04 (0.24)	0.05 (0.29)		
I hacked into someone’s account and stole personal information (e.g., through email or social networking accounts).	Exper	0.03 (0.24)	0.03 (0.24)	3.76	0.05
	Control	0.01 (0.14)	0.03 (0.25)		
I hacked into someone’s account and pretended to be them (e.g., through instant messaging or social networking accounts).	Exper	0.03 (0.25)	0.02 (0.21)	2.2	0.13
	Control	0.02 (0.17)	0.03 (0.23)		
I created a fake account, pretending to be someone else (e.g., through instant messaging or social networking accounts).	Exper	0.04 (0.25)	0.05 (0.28)	1.21	0.27
	Control	0.03 (0.21)	0.03 (0.23)		
I posted personal information about someone online.	Exper	0.03 (0.21)	0.03 (0.25)	0.01	0.96
	Control	0.02 (0.16)	0.03 (0.19)		
I posted embarrassing videos or pictures of someone online.	Exper	0.05 (0.30)	0.04 (0.26)	0.01	0.99
	Control	0.04 (0.24)	0.04 (0.25)		
I altered pictures or videos of another person that had been posted online.	Exper	0.05 (0.27)	0.04 (0.24)	0.93	0.33
	Control	0.05 (0.27)	0.04 (0.26)		
I excluded or ignored someone in a social networking site or Internet.	Exper	0.19 (0.55)	0.11 (0.42)	7.21	≥0.01 *
	Control	0.11 (0.38)	0.08 (0.33)		
I spread rumors about someone on the Internet.	Exper	0.08 (0.37)	0.07 (0.37)	0.10	0.75
	Control	0.06 (0.26)	0.05 (.28)		

Notes: * *p* < 0.05
